# Starch vs. tannin as biodegradable reagents for ultrafine hematite depression

**DOI:** 10.1038/s41598-024-65515-1

**Published:** 2024-06-25

**Authors:** Mehrdad Kordloo, Ahmad Rahmanian, Amirhossein Mohammadzadeh, Arash Tohry, Ali Rezaei, Saeed Chehreh Chelgani

**Affiliations:** 1 School of Mining Engineering, College of Engineering, University of Tehran, Tehran, 1439957131 Iran; 2 School of Chemical Engineering, The University of Queensland, Brisbane, QLD 4072 Australia; 3 Laboratory for Strategic Materials, Department of Chemical Engineering and Applied Chemistry, University of Toronto, 200 College Street, Toronto, ON M5S 3E5 Canada; 4 Mineral Processing, Department of Mining and Metallurgical Engineering, Yazd University, Yazd, 89195-741 Iran; 5 Minerals and Metallurgical Engineering, Swedish School of Mines, Department of Civil, Environmental and Natural Resources Engineering, Luleå University of Technology, SE-971 87, Luleå, Sweden

**Keywords:** Hematite, Tannin, Starch, Ultrafine, Flotation, Depression, Engineering, Chemical engineering

## Abstract

Enrichment of ultrafine liberated valuable minerals from their associated gangue phases is one of the emerging investigation topics within mineral processing and recycling. Using green flotation reagents and turning processes into eco-friendly systems is also one of the challenges in the green transition of ore beneficiation plants. Starch and Tanin as biodegradable depressants for hematite depression have been commercially used in various iron ore processing plants. However, their depression effects on ultrafine particles were not systemically assessed and compared. To fill this gap, this investigation examined the effects of starch, tannin, their mixtures (different ratios), and their different conditioning sequence on the floatability of ultrafine quartz and hematite (− 15 µm). Since the macromolecular polymer of these biodegradable depressants can bind particles together and flocculate them, turbidity analyses were used to assess their optimum ratio for hematite depression without affecting quartz floatability. Turbidity analyses provided a mixture of tannin and starch might enhance the flotation separation of quartz from hematite. Starch could flocculate ultrafine hematite particles, while tannin could disperse ultrafine quartz particles. Floatability experiments indicated that starch had the highest performance in hematite depression (lowest effect on quartz particles) compared to other conditions. Surface analyses (zeta potential and FTIR) proved floatability outcomes and highlighted starch had stronger adsorption on the hematite surface than tannin.

## Introduction

Through ore beneficiation processes, several particles would be over-ground (or liberated in quite ultrafine size fractions), resulting in the creation of ultrafine particles (below 20 µm), which typical upgrading techniques such as gravity^1^, magnetic, and flotation separation could not efficiently upgrade them^2^. In other words, ultrafine particles generate several challenges for upgrading processes^3–5^. Besides losing valuable minerals through process limitations and desliming, subjecting ultrafine particles to the tailing dams could create several environmental challenges. Fine and ultrafine particles deposited in the tailing dams can be dispersed by wind, settle down by rain or snow, and cause ecological issues^6^. On the other hand, an enormous increase in metals’ demands has been made meaningful processing of particles with ultrafine liberation degree. Thus, it would be essential to re-evaluate ultrafine processing regarding economic and environmental challenges through a green transition path.

Processing of finely disseminated hematite ores can be a particular example of the challenges mentioned above. Beneficiation of iron ore fines and slimes has noticeable benefits. It will result in better utilization of natural resources, a higher mine output in terms of economic view, and a reduction in the environmental impact of iron ore mining due to less residue material (tailings) for storage and disposal. Reverse flotation is the typical method for processing fine hematite-liberated particles where depressants substantially enhance the separation efficiency. Various depressants have been used for hematite surface deactivation through its upgrading by reverse flotation^7^. Some of these depressants are relatively toxic such as sodium cyanide. Starch and tannin are the most known biodegradable iron oxide depressants which markedly used on the industrial scale. It was reported that these polyphenolic (tannin)^8^ and polysaccharide (starch) depressants could depress iron oxides through various adsorption mechanisms and render hematite surface charge more negative and reduce their surface potential for collector adsorption^9^.

However, these depressants were hardly ever examined for the reverse flotation of ultrafine hematite ores, where the ultrafine particles have a strong propensity to enter the flotation froth product, resulting in poor separation efficiency. In addition, due to higher solubility than coarse particles, ultrafine particles tend to interfere with the flotation pulp chemistry and significantly affect their associated mineral surface properties^10–12^. As a novel comparative approach, this investigation has explored the potential of starch, tannin, and their mixture through the reverse flotation separation of ultrafine hematite from quartz. Quartz was selected since silicates are a prevalent gangue phase in iron oxide deposits^13^. It was reported that these biodegradable depressants, as macromolecular polymers, can bind particles together and flocculate them; therefore, their flocculation capabilities should be assessed through ultrafine particle processing^9^. Thus, the main aim of this investigation is to explore the process mechanisms of these two biodegradable depressants and compare their efficiency based on the minerals’ floatability. Different experiments (turbidity, flotation (single and mixed minerals), zeta potential and Fourier transform infrared spectroscopy (FT-IR)) were considered to highlight and strength the mechanism assessments.

## Material and methods

**Figure 1:**
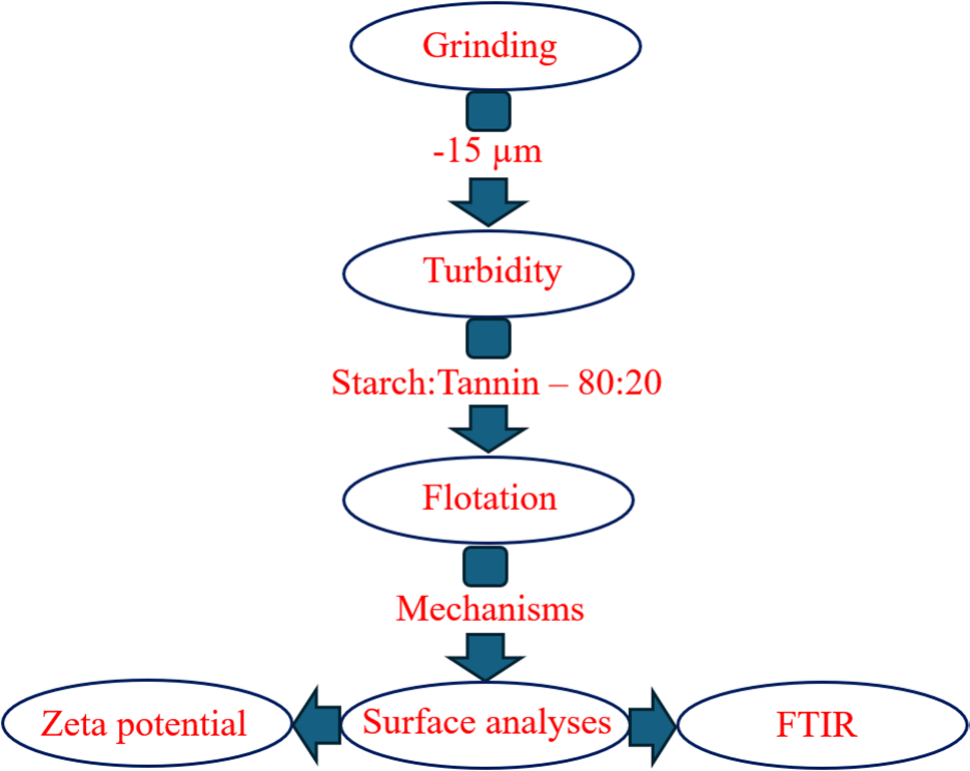
The flow diagram of experiments.

The summary of the experiments is illustrated in Fig. 1.

### Sample preparation

**Figure 2:**
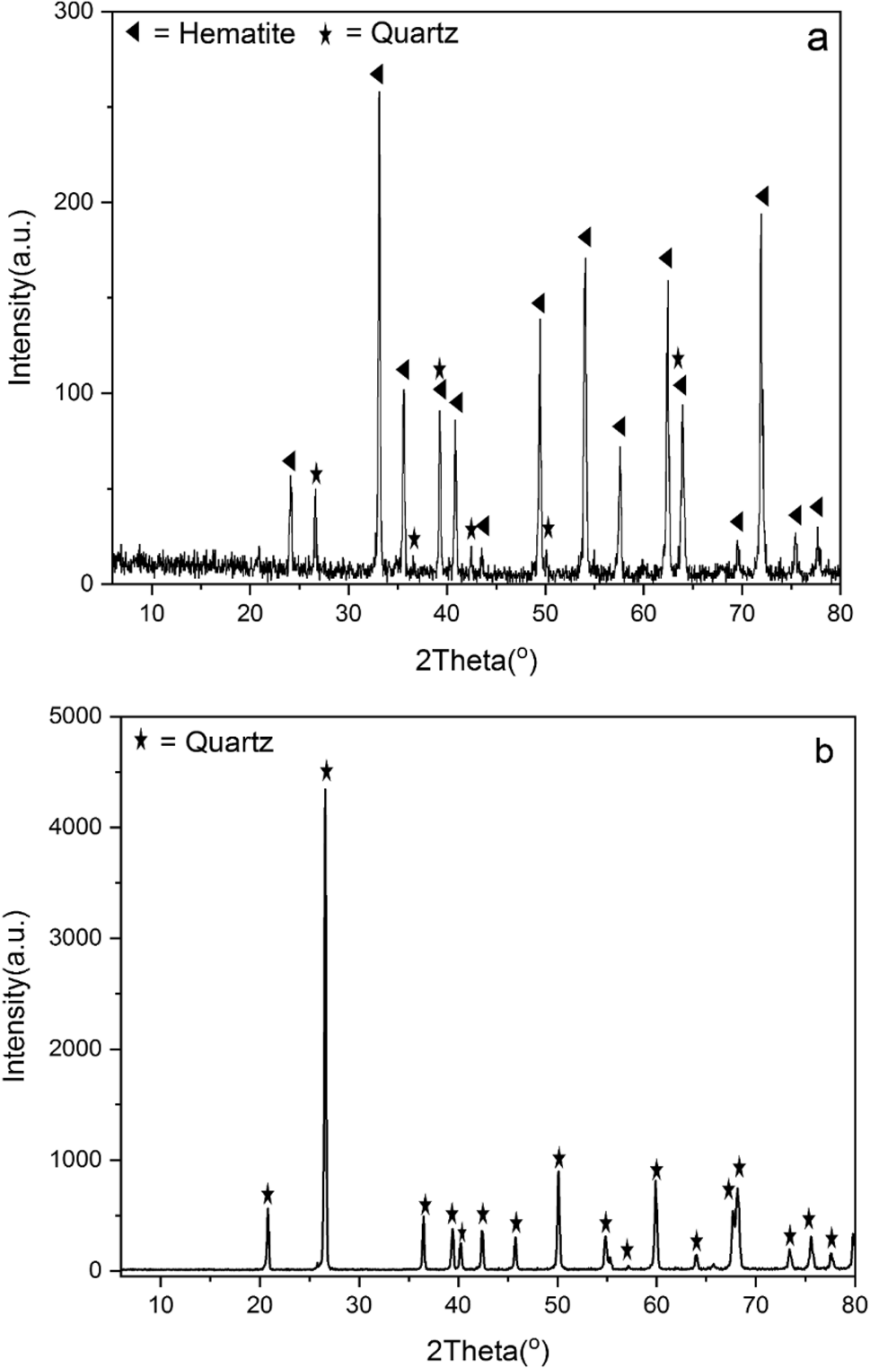
XRD patterns of (**a**) hematite and (**b**) quartz samples.

Pure hematite and quartz samples were obtained from the Akkahour Iron ore mine in Iran. Both mineral samples were divided into two sections, and one part was saved. The results of X-ray fluorescence (XRF) and X-ray diffraction (XRD) are shown in Table 1 and Fig. 2, confirming the high purity of the quartz and hematite. A cone crusher first crushed both samples to less than 10 mm. Then, they were milled to below 25 µm using a ball mill. The milling operation was a stepwise process that continued for only 10 min each time to prevent particles from sticking together again. After every 10 min of milling, particles below 25 µm were sieved and removed. The samples underwent crushing and grinding procedures; the size fraction of − 15 μm was used for subsequent turbidity, flotation, zeta potential, and FT-IR measurements.

**Table 1:** Chemical composition of hematite and quartz samples (wt%).

Minerals	XRF analysis (%)										
	Fe_2_O_3_	LOI	SiO_2_	TiO_2_	Al_2_O_3_	P_2_O_5_	BaO	K_2_O	Na_2_O	MgO	MnO
Hematite	95.55	0.54	2.81	0.014	0.48	0.05	0.12	0.07	0.05	0.08	0.16
Quartz	1.00	0.17	97.75	0.07	0.75	–	–	0.24	–	–	–

### Turbidity

Turbidity tests (Table 2) were performed by a Thermo Scientific turbidity meter (Model: AQUAfast II Orion AQ2010) to assess the effectiveness of starch, tannin, and their mixtures on the flocculation of hematite and quartz. In these experiments, the flocculant was either tannin, starch, or a mixture of both. The turbidity tests were conducted in 100 mL graduated cylinders under specified conditions: pH 9, a solid weight ratio of 1%, and a designated flocculant concentration. Different concentrations of tannin, starch, and their mixtures (in various ratios) were used.

**Table 2:** The concentration of depressants for turbidity tests (single and mixed reagents).

Single reagents	Concentration (PPM)						
Starch	0	50	150		300		
Tannin	0	50	150		300		

Mixed (100 ppm)	Ratio (%)						
Starch	0	100	80	60	40	20	0
Tannin	0	0	20	40	60	80	100

Tannin was dissolved in distilled water to prepare the tannin solution, and additional distilled water was added to achieve a final volume of 100 mL. In the case of starch, due to its limited solubility in water, a meticulous approach was taken to ensure precise concentration was achieved. Initially, 1 g of corn starch was dissolved in a 10 mL solution of NaOH (10%) before being brought to a final volume of 100 mL using distilled water.

After adjusting the pH as required, the sample was introduced into the solution and subjected to agitation. At room temperature, the suspension was stirred by a magnetic agitator at 500 rpm for three minutes. After preparation, the solution remained stationary for three minutes. Subsequently, a precipitation time of 5 min allowed for the flocs’ formation. Experiments were repeated 3 times, and the average was considered. Sampling was carefully performed from the uppermost 10 mL of the cylinders, ensuring representative data collection. Various tests were performed to measure the flocculation ability of tannin and starch.

### Floatability

Flotation experiments were conducted carefully to effectively compare the performance of tannin, starch, and their mixture on the floatability of ultrafine quartz and hematite. Experiments were performed in a Hallimond tube. In these experiments, 1 g of the sample was carefully poured into an 80 mL container with ventilation set at 100 mL/min and an impeller speed of 650 rpm. Additionally, a specific procedure was followed for the flotation reagents: initially, 1 g of the sample was conditioned in water for 1 min, followed by adding the depressant with 3 min of conditioning. Subsequently, Lilaflot®811M was introduced into the mixture and conditioned for more 2 min. As a quartz collector, Lilaflot has been used in various studies for reverse iron ore flotation^14,15^. Lilaflot proved to be a selective collector that can produce a high-grade quartz concentrate with high metallurgical recovery^16^. The tests took place in a controlled environment using the Lilaflot. The system's pH was carefully set to 9 for the quartz flotation tests, ensuring an optimal condition. Multiple concentrations of Lilaflot (15, 30, 75, and 90 ppm) were assessed. A consistent stirring time of 2 min was maintained to facilitate proper mixing and interaction between the flocculant and the particles. The flotation process was initiated by introducing air into the flotation cell. Likewise, parallel flotation tests were conducted on hematite, replicating the experimental setup and conditions employed for the quartz flotation tests. The recovery of hematite was evaluated for each Lilaflot concentration. Based on the quartz and hematite recovery results, the Lilaflot concentration that yielded the highest quartz recovery (lowest hematite recovery) was identified as the optimized concentration. These tests were repeated five times to ensure reliability. After determining the optimized concentration of Lilaflot®811M for flotation tests, additional experiments were conducted to assess the depressant performance of tannin and starch. Experiments were performed under the optimized conditions. The depressants’ concentration (tannin and starch) and their mixture were selected based on the turbidity results. Different sequences of adding a tannin-starch mixture were considered.

### Zeta potential

Zeta potential measurements were conducted to understand better the surface charge characteristics of hematite and quartz particles through various flotation conditions. The zeta potential measurements were conducted both in the presence and absence of flotation reagents (tannin, starch, their mixture, and Lilaflot) at pH 9, based on the optimal flotation conditions. Through the assessment, particles were suspended in a well-suited electrolyte solution, and their zeta potential was determined. This involved employing a reliable and appropriate zeta potential measurement instrument. Zeta potential measurements were executed utilizing a Malvern zeta sizer (nano–ZS90). 50 ± 1 mg of the mineral sample was gently introduced into a 100 mL solution to create the mineral sample suspension. The solution's pH and reagent concentrations were thoughtfully adjusted for each distinct experiment. The suspension was subjected to magnetic stirring for 3 min to ensure proper conditioning. The pH value was monitored continuously and maintained throughout the conditioning phase. This was accomplished by embedding a pH-meter electrode within the solution and a pH modifier-enhanced needle to sustain constant pH levels. The suspension was then allowed to stand for approximately 5 min, allowing the particles to settle. The supernatant, comprising around 3 mL, was subsequently extracted and employed for zeta potential measurement. These experiments were consistently executed under controlled conditions of 22 ± 1 °C. The presented results are the average derived from five measurements.

### FTIR

Fourier transform infrared spectroscopy (FTIR) was employed to analyze the molecular environment and composition of the tannin, starch, and their mixture functional groups on the surface of pure minerals before and after conditioning. The FTIR studies were conducted using a Bruker Equinox-55 FTIR analyzer manufactured in Germany, covering a scanning range from 400 to 4000 cm^–1^. For the analyses, 1.0 g of each pure mineral was introduced into an aqueous solution containing the conditioners at a concentration of 100 mg/L. Then, samples were conditioned for 2 h while the pH of the solution was maintained at around 9. The particles underwent filtration and subsequent drying at ambient temperature for 24 h. Samples were combined with potassium bromide at a weight ratio of 1% at ambient temperature, and the spectra were afterward measured at a resolution of 4 cm^–1^^17^. The spectral analysis of unaltered mineral samples was conducted before any intervention by the conditioners, aiming to facilitate comparative analysis.

## Result and discussion

### Turbidity

**Figure 3:**
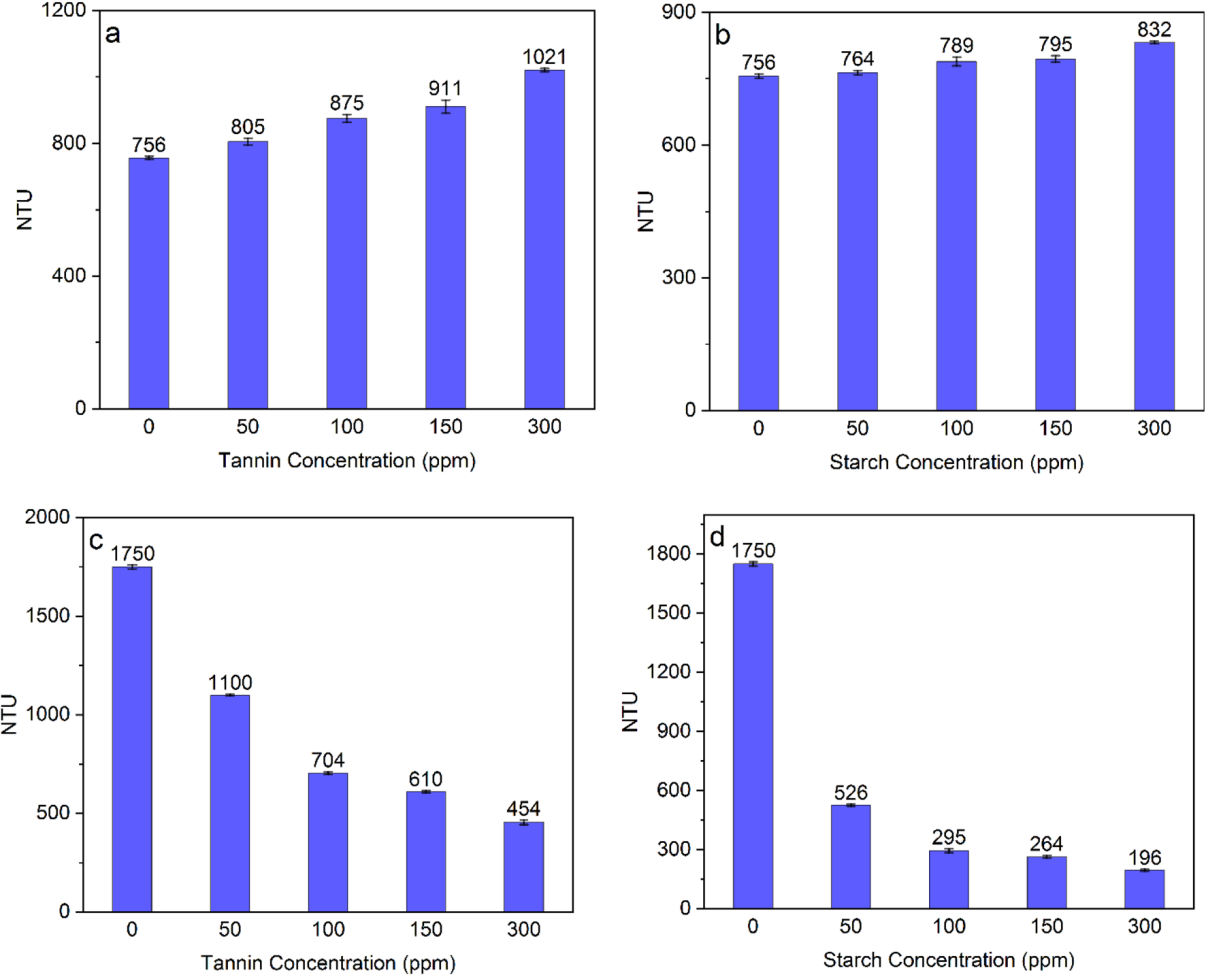
Turbidity analyses of (**a**) quartz-tannin, (**b**) quartz-starch, (**c**) hematite-tannin, (**d**) hematite-starch systems.

Turbidity has the potential to provide insights into the degree of dispersion or aggregation exhibited by powders within a liquid medium. Understanding these dual occurrences can play a pivotal role in elucidating the intricate mechanisms that govern the interplay between mineral particles and reagents within an aqueous solution^8,18^. Turbidity tests were conducted to understand the flocculation properties of samples conditioned with tannin, starch, and their mixture. Turbidity test results (Fig. 3a) indicated that starch, even in low concentration, could reduce hematite turbidity, which has been addressed in other investigations as well^19,20^. Tannin showed the same trend as starch generated; however, starch caused significantly lower hematite turbidity than tannin in their various concentrations (Fig. 3b vs. a). Turbidity test results on quartz (Fig. 3b and d) showed both starch and tannin had a reverse impact on its flocculation process. Tannin tended more than starch to increase quartz turbidity (Fig. 3b and c). Tohry indicated that tannin had a dispersion effect on the fine quartz particles while aggregating hematite particles^8^. As a comparison, starch resulted in a more beneficial influence on the aggregation of hematite particles, whereas tannin proved to be the superior agent for dispersing quartz particles. It has been shown that starch can be a selective flocculant for hematite^21^, while it can reduce the quartz inclusion throughout the flocculation process^22^. These outcomes highlighted the possibility of increasing the flotation separation efficiency of ultrafine hematite from quartz by mixing starch and tannin and gaining their synergic effects (flocculating ultrafine hematite and dispersing ultrafine quartz particles).

**Figure 4:**
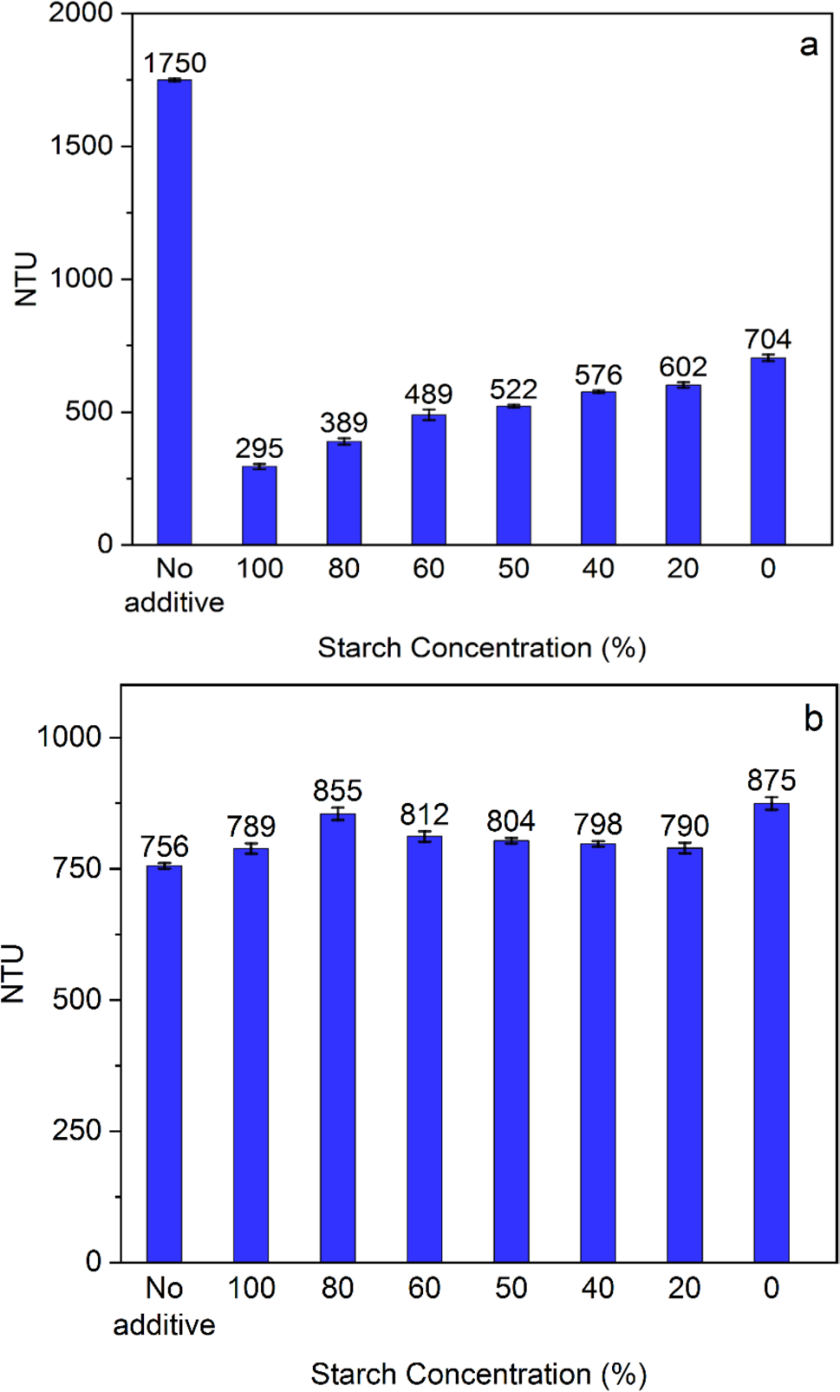
Turbidity tests by the mixture of starch and tannin with (**a**) hematite and (**b**) quartz.

Various starch-tannin ratios were examined to explore that scenario (Fig. 4), while the total regent concentration was 100 ppm. As anticipated, the inclusion of tannin in the mixture is associated with a decrease in the hematite flocculation (the turbidity value was raised when the tannin percentage in the mixture was increased) (Fig. 4a). However, the mixture with 80% starch and 20% tannin in the hematite test showed approximately a close turbidity value as 100% starch (295 vs. 389 NTU). The same scenario could be observed for quartz (Fig. 4b), where 80% starch and 20% tannin resulted in a similar turbidity value as 100% tannin (855 vs. 875 NTU). In other words, this specific combination (80% starch and 20% tannin) demonstrated a potential synergistic interaction on both hematite and quartz ultrafine particles. For assessing the possible effects of this synergistic interaction on the separation of ultrafine hematite from quartz, an 80/20 ratio was considered for the flotation experiments to compare with pure starch and tannin performance.

### Floatability

#### Collector

**Figure 5:**
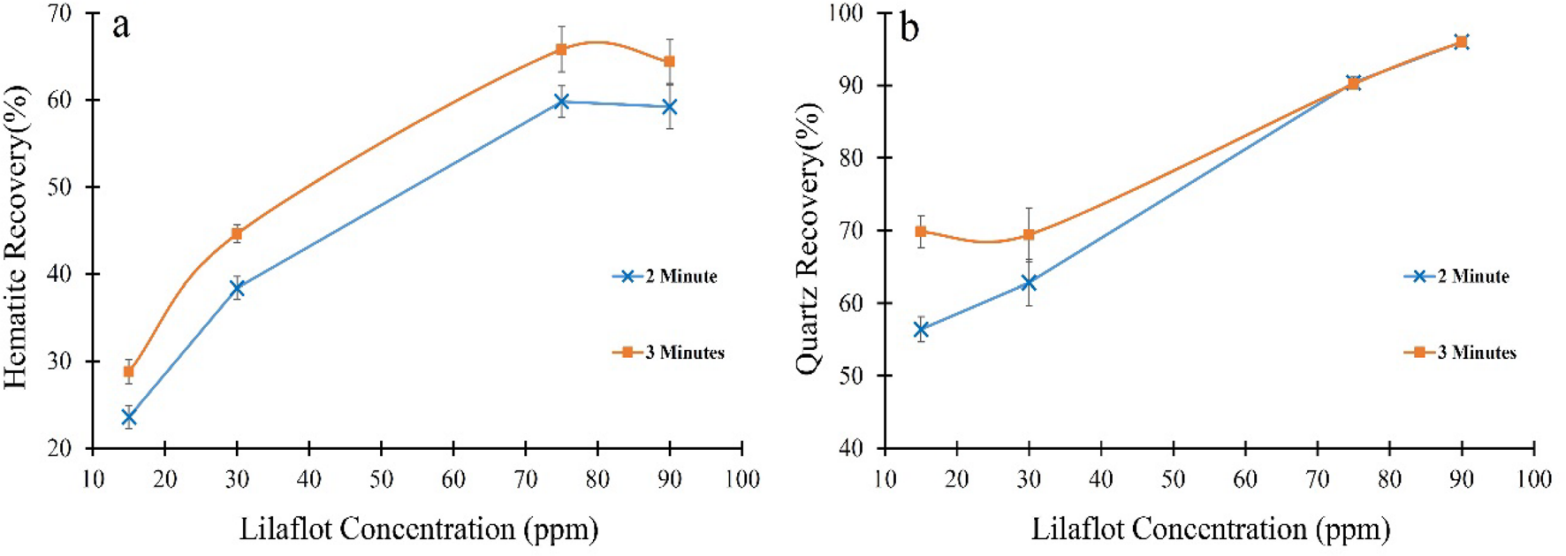
Flotation results of (**a**) Hematite and (**b**) quartz with different Lilaflot concentrations and skimming time.

Single mineral flotation was conducted to find the optimum collector (Lilaflot) concentration for ultrafine quartz and hematite floatability. The result of floatability tests with different time skims (Fig. 5) indicated that the recovery of both minerals was raised by increasing the collector concentration. 90 ppm Lilaflot resulted in approximately ~ 65% hematite recovery after 3 min process (Fig. 5a). It was reported that “although Lilaflot is a selective collector for quartz, its high concentration could also increase the recovery of iron oxides’’^23^. These outcomes highlighted the importance of using depressants for the reverse flotation separation of hematite from quartz. Since approximately the whole quartz was recovered after 2 min (Fig. 5b), and the recovery of hematite was lower during 2 min, this time skim, and 90 ppm Lilaflot were selected as the optimum floatability conditions for assessing starch and tannin depression efficiency.

#### Depressants

**Figure 6:**
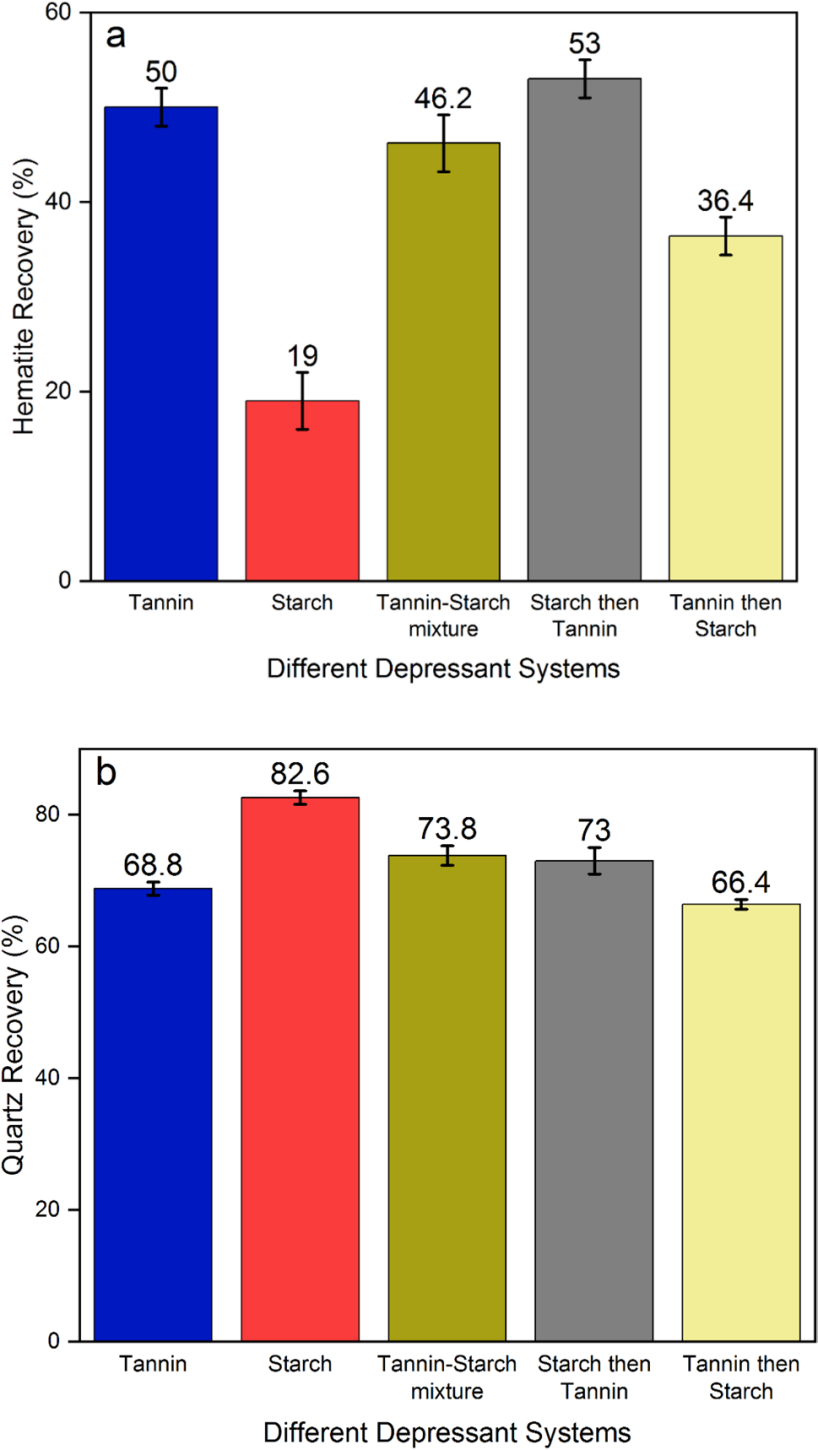
Flotation of (**a**) hematite and (**b**) quartz with 100 ppm of different depressant systems, 90 ppm of Lilaflot, and 2 min skimming time.

To explore the effects of depressants (starch and tannin) on the floatability of both minerals (hematite and quartz), various scenarios were assessed: starch, tannin, and their mixture (80/20) with different sequences. For different sequence approaches, samples were conditioned simultaneously but in various orders: first with starch (80 ppm) and then tannin (20 ppm), first tannin and then with starch, and with starch and tannin together. After each conditioning, the sample was treated with 90 ppm Lilaflot. Floatability test results indicated (Fig. 6) that starch had superior depression efficiency by achieving the lost hematite floatability and highest quartz recovery (reverse flotation) compared to all other conditions. None of the mixture conditions could provide promising metallurgical responses. It is important to note that starch can depress both quartz and hematite particles. However, compared to hematite, starch adsorption on quartz is limited in alkaline pH^24,25^. Tannin slightly reduced quartz floatability. The physical interaction between the silanol groups on the quartz surface and the tannin molecules may be responsible for the minor depression effect of tannin on the quartz surface^18,26,27^.

### Zeta potential

**Figure 7:**
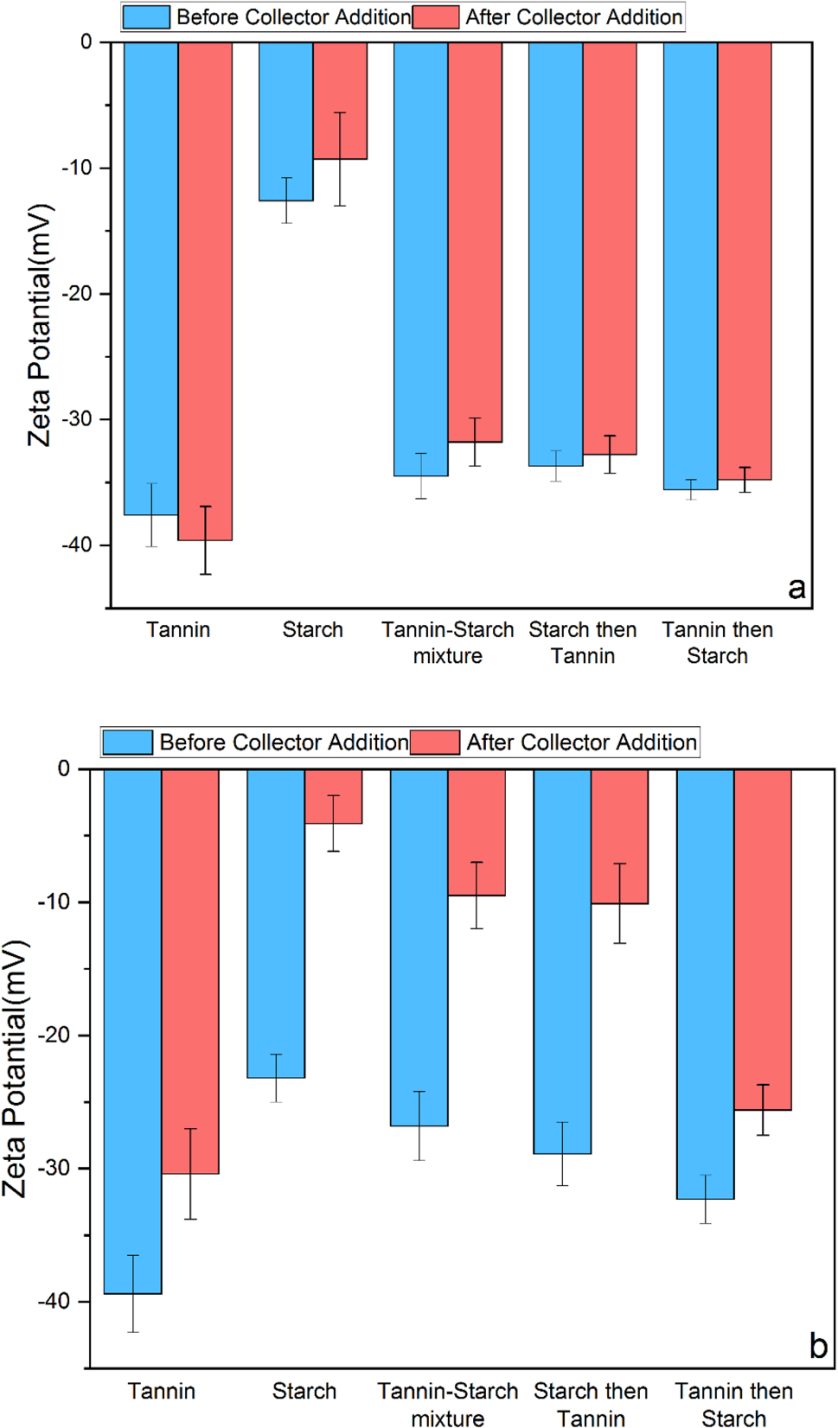
Zeta potential of (**a**) hematite and (**b**) quartz before and after collector addition with different depressant conditionings.

It was reported that the Lilaflot adsorption could decrease the zeta potential on the surface of iron oxides and quartz^15^. The zeta potential measurements on the surface of minerals after various conditioning indicated (Fig. 7) that the zeta potential variation on the hematite surface treated only with starch had the minimum variation after conditioning with the collectors compared to other conditions. In other words, conditioning hematite with starch minimized the Lilaflot adsorption on its surface (Fig. 7a). All other conditioning with mixtures showed a similar behavior as treatment by tannin on the hematite surface. On the other hand, quartz samples treated through various conditionings by depressants showed a similar zeta potential pattern (Fig. 7b). These outcomes could be translated to the minimum effect of depressant conditioning on the collector adsorption rate for the treated quartz surfaces. However, starch showed the minimum effects on quartz collector adsorption. These results are aligned with the flotation outcomes (Fig. 6).

### FTIR

**Figure 8:**
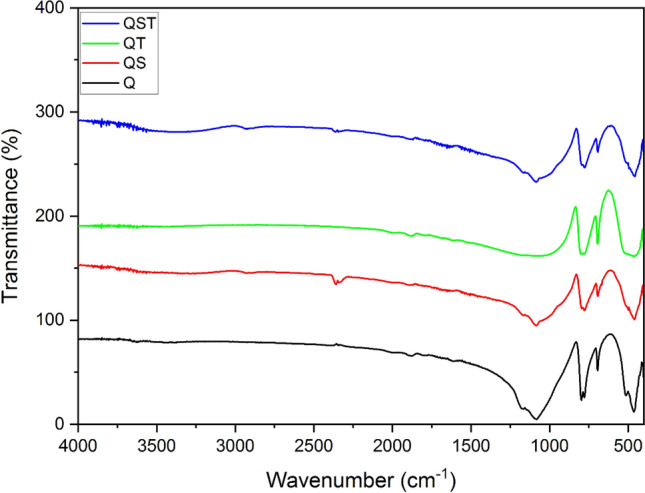
FT-IR spectra of Quartz. Q: pure quartz QS: quartz treated by starch, *QT* quartz treated by tannin, *QST* quartz treated by starch and tannin mixture.

**Figure 9:**
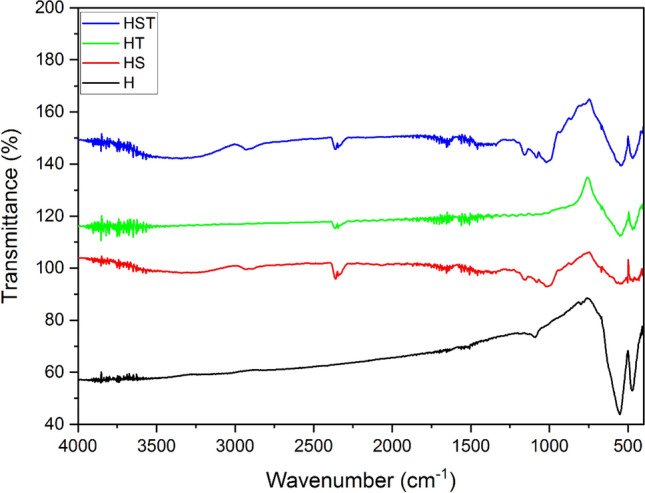
FT-IR spectra of Hematite; *H* pure hematite, *HS* hematite treated by starch, *HT* hematite treated by tannin, *HST* hematite treated by starch and tannin mixture.

Since various orders of conditioning with starch and tannin mixtures did not show any specific improvement in their depression efficiency, FTIR analyses were only conducted on pure minerals, minerals treated individually with tannin and starch, and one mixture (tannin and starch together (80/20)) (Figs. 8 and 9). The FTIR spectrum analysis of quartz exposed to tannin treatment revealed interactions marked by the gradual decrease in absorption bands' intensity from 4300 to 2400 cm^–1^, suggesting persistent interactions, potentially due to hydrogen bonding and van der Waals forces. A discernible peak at around 2350 cm^–1^ indicated a specific interaction between tannin's functional groups and the quartz surface. Another prominent peak at 1750 cm^–1^ suggested a chemical shift, possibly due to ester or carboxylic acid linkages between tannin and quartz. Tannin's presence in the treated spectrum can be confirmed by a notable peak at 1080 cm^–1^, suggesting alterations in Si–O–Si bending vibrations^28^. In contrast, FTIR spectra of quartz exposed to starch treatment showed distinct interactions. The initial transmission increased at approximately 4300 cm^–1^, indicating interactions between starch and the quartz surface. A subtle peak around 2350 cm^–1^, followed by a return to the baseline, suggested a complex starch-quartz interaction. The spectrum of quartz treated with starch exhibited a significant peak at 1100 cm^–1^, indicative of hydrogen bonding between starch's hydroxyl groups and quartz's silanol groups. Variations in wavenumbers at 850 cm^–1^ and 600 cm^–1^ implied complex surface bonding changes, potentially involving Si–O–C or Si–O–OH bonds^29,30^.

Quartz exposed to an 80/20 mixture of tannin and starch displayed a unique interaction pattern. Initial transmission and vibrational modes confirmed the presence of the mixture on the quartz surface. A decline in transmission, followed by peaks at 3000 cm^–1^ and 2400 cm^–1^, indicated altered surface bonding and novel contacts. A significant peak at 875 cm^–1^ suggested a strong interaction between the mixture and the quartz surface. Oscillations in the 750 cm^–1^ to 600 cm^–1^ range indicated intricate interactions, likely involving various functional groups from both tannin and starch compounds. Fundamentally, the differences in absorption bands and shifts observed in the tannin, starch, and mixture treatments provide insights into their unique interactions with quartz, likely attributed to hydrogen bonding, chelation, coordination, and surface adsorption.

FTIR spectra of hematite exposed to starch exhibit vibrational frequencies from 3950 to 3450 cm^–1^, indicating potential hydrogen bonding interactions between starch hydroxyl groups and hematite surface silanol groups. Peaks at 3300 and 3200 cm^–1^ correspond to O–H stretching vibrations in starch and hematite surface hydroxyl groups, possibly involving various iron-coordinated OH groups, which can be seen in all spectra. A signal at 2400 cm^–1^ suggested additional interactions, potentially related to carbonyl groups. These findings indicated coordination between starch carbonyl oxygen and surface metal ions. A spectral range from 2000 to 1300 cm^–1^ demonstrated intricate interactions, likely involving diverse functional groups. Peaks at 1640 and 1540 cm^–1^ correspond to bending and stretching vibrations of O–H groups in starch. The peak at 750 cm^–1^ showed novel surface complexes or contacts, possibly involving hydrogen bonding between starch and surface silanol groups. The declining transmission at 575 cm^-1^, followed by an increase, suggested a mix of surface bonding and rearrangements, encompassing hydrogen bonding and potential coordination^29,30^.

FTIR spectra of hematite exposed to tannin show slight transmission elevation up to 900 cm^–1^, indicating interactions between tannin's functional groups and hematite. A spectral region from 4000 to 3350 cm^–1^ demonstrated robust interactions, possibly hydrogen bonding between tannin hydroxyl groups and hematite surface silanol groups. A signal at 2400 cm^–1^ indicated supplementary interactions related to carbonyl groups. These interactions may involve coordination between tannin carbonyl groups and surface metal ions. A spectral range from 2000 cm^-1^ to 1300 cm^-1^ showed diverse functional group interactions. Peaks at 1640 and 1540 cm^–1^ correspond to bending and stretching vibrations of O–H groups in tannin. A peak at 750 cm^–1^ potentially indicated novel surface complexes or contacts, likely involving hydrogen bonding between tannin and surface silanol groups. The steady decline at 575 cm^–1^, followed by an increase, suggested a combination of surface bonding and rearrangements, encompassing hydrogen bonding and potential coordination^30,31^.

FTIR spectra of hematite exposed to a tannin-starch mixture reveal tiny segments dispersed throughout the spectrum, suggesting both tannin and starch involvement in surface interactions. A vibration range from 1950 to 1350 cm^–1^ demonstrated intricate interactions involving several functional groups from both compounds. Peaks at 1640 and 1540 cm^–1^ correspond to O–H bending and stretching vibrations in both tannin and starch. Two minor peaks followed by a decline toward 950 cm^–1^ indicated distinct surface complexes or hydrogen bonding interactions between tannin and starch functional groups and hematite surface silanol groups. The spectrum highlights complex coordination, hydrogen bonding, and surface rearrangements resulting from the combined impact of both adsorbents on hematite surfaces^8^. FTIR results generally highlighted a stranger interaction of starch with hematite than tannin and the mixture.

## Discussion

Based on the turbidity results, it was anticipated that a mixture of tannin and starch mixture could lead to a higher flotation efficiency, as their mixture could flocculate hematite particles and disperse quartz particles properly. However, flotation experiments and surface assessments indicated a failure of such a hypothesis. At alkaline pH, the hematite surface is hydrophilic and well-wetted by water because surface hydroxylation occurs at these pH levels^32^. Therefore, depressants must minimize the collector absorption on the hematite surface. Starch macromolecules, rich in hydrophilic OH^-^ groups from their D-glucose monomers, can extend in solution and act as mineral particle bridges, functioning as flocculants. While initially attributed to hydrogen bonding between starch and hematite, the selective adsorption on hematite occurred mainly due to hydroxylated Fe-sites on the hematite particles^33,34^. However, it has been shown that a high starch concentration (more than 100 ppm) can also depress quartz particles^35^. Quartz is also hydrophilic at 10.5 pH and does not bind to bubbles, and it has been shown that either maize 50 ppm of starch has no contact at all with the quartz surface or that there is interaction (adsorption) but that the quartz surface retains its hydrophilic properties^36^. In other words, studies have shown that starch exhibited a limited affinity for certain minerals, such as quartz. However, as a macromolecular polymer, starch can facilitate particle binding, resulting in a mild depression of quartz^37,38^. Moreover, ultrafine particles have more surface activity. Hence, quartz particles may interact with starch in lower concentrations. Based on the result of this study, starch can flocculate and depress hematite particles efficiently. However, in the case of quartz, it seems that the interaction of quartz and starch only resulted in dispersion and had no effect on the quartz depression. Notably, the FTIR spectra of quartz exposed to starch demonstrated distinct interactions, with an initial increase in transmission, suggesting a connection between starch and the mineral surfaces. As the zeta potential result showed, all depressants had interaction with the quartz surface, but starch resulted in a lower surface charge, which resulted in lower dispersion and higher collector adsorption than other depressants. This is further supported by the lower surface charge observed for starch, which, as indicated in the FTIR results, led to reduced dispersion compared to other conditions.

It has been reported that tannin interacts with quartz and iron-oxide particles^39^. A strong non-electrostatic or chemical contact may cause the substantial adsorption of tannin molecules onto hematite particles. On the other hand, its adsorption on quartz particles is mostly physical^9^. It has been stated that tannin may selectively depress hematite by more than 90% with a minimal impact of only 8% on quartz floatability^8^. However, it has also been demonstrated that tannin can disperse quartz-containing clay minerals in the presence of sodium hydroxide^40^. Engwayu indicated that lignosulfonate dispersed silicate particles at both high and low pH levels^5^. This investigation indicated although tannin worked better than starch in dispersing quartz particles, the flotation results showed that starch is a better depressant than tannin. The zeta potential results showed that tannin could not, like starch, prevent collector absorption.

Moreover, XPS analysis from other investigations indicated the starch adsorption onto hematite surfaces could result in notable shifts in the C–OH components toward higher binding energies, indicating strong attachment through COO − and C–OH moieties^41^. XPS outcomes confirmed that the Fe 2p binding energies on hematite surfaces change upon the addition of starch-based depressants, suggesting significant chemical interaction primarily between Fe(III) and the starch molecules^42^. It was highlighted that the starch adsorption might introduce new components such as C–C/C–H, C–OH, and O–C–O/C=O on the hematite surface, which are associated with the chemisorption of starch molecules on FeO–OH and Fe–OH surface sites^43,44^. DFT analysis reveals that starch adsorbed on the hematite surface covers a large area, encapsulating multiple hematite particles and making the boundaries between them visible. These observations are aligned with the role of corn starch in promoting the flocculation of fine-grained hematite particles^45^. In addition, density functional theory (DFT) calculation studies pointed that the adsorption strength of starch fragments on the hematite hydrated surface is greater compared to that on quartz^46^.

In general, XPS analysis demonstrated that starches chemisorb onto hematite surfaces rather than quartz surfaces^42^. It has been shown that no chemisorption occurs between starch molecules and Si atoms on quartz, suggesting that starch adsorption on the quartz surface is likely due to hydrogen bonding with silanol groups^44^. This conclusion is supported by XPS spectra of pargasite, which show that starch could adsorb on the amphibole surface through hydrogen bonding with surface hydroxyls, similar to the quartz surface, and also forms surface chemical complexes with metal atoms, as observed in magnetite surfaces^24^. It was documented that XPS analyses of starch-treated quartz and hematite, strongly corroborating findings from flotation experiments and zeta potential measurements^47^.

However, no study was reported on XPS and DFT analyses of tannin and its mixture for a hematite-silicate system. Thus, future studies can focus on various areas to expand on these findings. Flotation experiments on actual mine ores would determine these biodegradable depressants' practical application and efficacy. Furthermore, studying the underlying chemistry of tannin and starch co-adsorption with XPS and DFT calculation might reveal more information on their synergistic effects.

## Conclusion

The depression performance of tannin and starch as biodegradable depressants on ultrafine (− 15 µm) hematite and quartz floatability was examined in various conditions. Turbidity analyses indicated that a mixture of tannin and starch might have a synergic effect through the reverse flotation separation of hematite from quartz. It was illustrated that starch can act as a flocculant for ultrafine hematite in the mixture, and tannin may disperse ultrafine quartz particles. Increasing starch concentration generally had a sharper effect on the hematite turbidity reduction than tannin, while tannin could increase the quartz ultrafine particles’ turbidity higher than hematite. Turbidity results showed a mixture with 80% starch and 20% tannin could provide close turbidity results for each of these flotation reagents and for both minerals. Floatability test results indicated that 2 min conditioning with a 90 ppm collector (Lilaflot) would be the optimum condition. Adding depressants with various conditions (starch (80 ppm), tannin (20 ppm), and their mixture (80/20)) with different treatment orders (first with starch and then tannin first tannin and then with starch, and with starch and tannin together) demonstrated that starch had superior depression efficiency by achieving the lost hematite floatability and highest quartz recovery (reverse flotation) compared to all other conditions. Surface analyses (zeta potential measurement and FTIR) highlighted particles conditioned with starch had the highest stability on their surface charges while having the lowest effect on collector adsorption on the quartz surface compared to all other conditions.

## Data Availability

The datasets generated and/or analyzed during the current study are not publicly available due to NDAs signed with companies but are available from the corresponding author upon reasonable request.
